# HSPB8-BAG3 chaperone complex modulates cell invasion in intrahepatic cholangiocarcinoma by regulating CASA-mediated Filamin A degradation

**DOI:** 10.1080/15384047.2024.2396694

**Published:** 2024-08-31

**Authors:** Bo Shu, Yu Wen, Ronghua Lin, Chao He, Cailan Luo, Fazhao Li

**Affiliations:** a Department of General Surgery, The Second Xiangya Hospital, Central South University, Changsha, Hunan Province, China; b Department of General Surgery, Huichang County People’s Hospital, Huichang, Jiangxi Province, China; c Department of Hospital Nursing, Huichang County People’s Hospital, Huichang, Jiangxi Province, China

**Keywords:** HSPB8, BAG3, Filamin A, intrahepatic cholangiocarcinoma, CASA

## Abstract

The incidence of intrahepatic cholangiocarcinoma (ICC) is steadily rising, and it is associated with a high mortality rate. Clinical samples were collected to detect the expression of HSPB8 and BAG3 in ICC tissues. ICC cells were cultured and transfected with plasmids that overexpressed or silenced specific genes to investigate the impact of gene expression alterations on cell function. qPCR and Western blot techniques were utilized to measure gene and protein expression levels. A wound healing assay was conducted to assess cell migration ability. The Transwell assay was used to assess cell invasion ability. Co-IP was used to verify the binding relationship between HSPB8 and BAG3. The effects of HSPB8 and BAG3 on lung metastasis of tumors in vivo were verified by constructing a metastatic tumor model. Through the above experiments, we discovered that the expressions of HSPB8 and BAG3 were up-regulated in ICC tissues and cells, and their expressions were positively correlated. The metastatic ability of ICC cells could be promoted or inhibited by upregulating or downregulating the expression of BAG3. Furthermore, the HSPB8-BAG3 chaperone complex resulted in the abnormal degradation of Filamin A by activating autophagy. Increased expression of Filamin A inhibits the migration and invasion of ICC cells. Overexpression of HSPB8 and BAG3 in vivo promoted the lung metastasis ability of ICC cells. The HSPB8-BAG3 chaperone complex promotes ICC cell migration and invasion by regulating CASA-mediated degradation of Filamin A, offering insights for enhancing ICC therapeutic strategies.

## Introduction

Intrahepatic cholangiocarcinoma (ICC) is a rare type of primary liver tumor with a high metastatic potential. The incidence and mortality rates of ICC have been increasing in recent years.^1^ ICC’s high mortality rate is attributed to two main factors. Firstly, there are a few specific signs that can be used to diagnose ICC when it occurs. Secondly, its strong metastatic potential makes it challenging for physicians to completely remove the lesion using the most effective surgical treatment.^2^ Numerous scholars are actively investigating the key molecules involved in the recurrence of ICC metastasis. At present, it has been found that ICC metastasis is mainly influenced by activating various pathways in cancer cells to regulate cell metabolic reprogramming, epithelial-mesenchymal transition (EMT), autophagy, and immune escape.^3^ However, further research is necessary to improve the prognosis of ICC patients and identify novel therapeutic targets.

The heat shock protein B8 (HSPB8), a type of chaperone involved in chaperone-assisted selective autophagy (CASA), has been shown to play a significant role in the invasive metastasis of cancer cells.^4^ Previous research has indicated that the expression level of the HSPB8 protein is strongly associated with the invasiveness of cancer tissue and lymph node metastasis in gastric cancer.^5^ Furthermore, as a positive regulator of TGF-α-induced tumor cell metastasis, HSPB8 could promote the advancement of ovarian cancer.^6^ Our previous study found that HSPB8 is overexpressed in ICC, which promotes EMT in ICC cells.^7^ Nevertheless, the potential mechanism of HSPB8‘s role in invasive metastasis of ICC has not been identified.

BCL2-associated athanogene 3 (BAG3) is a member of the BAG (Bcl-2-associated anti-apoptotic gene) family. It acts as a co-molecular chaperone of HSPB8 to mediate CASA.^8^ In gastric cancer, BAG3 enhances the proliferation and invasion of cancer cells through the ERK/EGR1 pathway.^9^ In colorectal cancer, the overexpression of BAG3 not only promotes cell growth, migration, and invasion but also enhances the resistance of cancer cells to the chemotherapeutic drug 5-FU, thereby improving tumor survival.^10^ BAG3 plays a significant role in various types of cancers, but its role in ICC has not been investigated.

Recent studies have demonstrated that Filamin A is one of the prominent substrates of CASA. It has been reported that the HSPB8/BAG3 pathway mediates CASA and induces autophagic degradation of denatured Filamin A under high cellular tension.^8^ A study has demonstrated that silencing Filamin A in colorectal cancer promotes cell migration and invasion, thereby facilitating cancer progression.^11^ Another study reported that Filamin A is downregulated in breast cancer, which suppresses the migration, invasion, and metastasis of breast cancer cells.^12^ A recent study found that Filamin A is cleaved by calpain to produce a 90 kDa fragment. This fragment is then transferred to the nucleus where it inhibits the transcription of downstream genes, thereby mediating ICC cell metastasis.^13^ However, its role in ICC needs to be further explored experimentally.

In summary, we propose the hypothesis that the HSPB8-BAG3 molecular chaperone complex promotes ICC cell invasion by regulating CASA-mediated Filamin A degradation. This will provide a strong foundation for discovering and implementing new targets for treating ICC.

## Results

### HSPB8 and BAG3 were highly expressed in ICC

**Figure 1.**
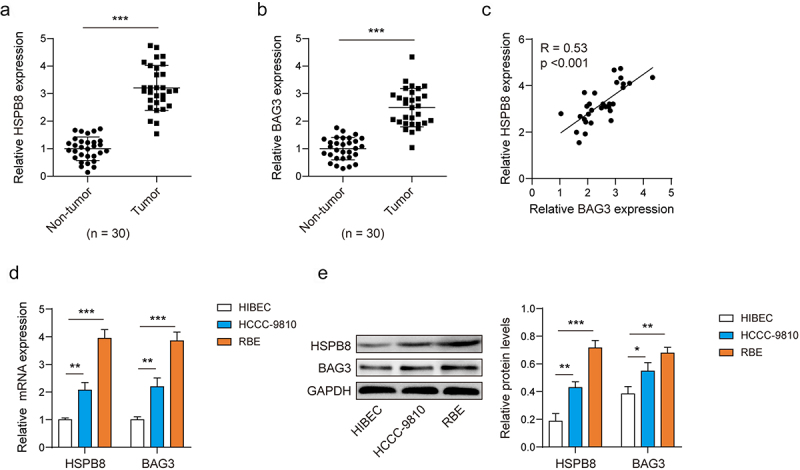
HSPB8 and BAG3 were highly expressed in ICC.

The expression levels of HSPB8 and BAG3 were found to be abnormally elevated in ICC tissues (Figure 1(a,b)). Based on this similar variation, we conducted a Pearson correlation analysis to verify the association between the two genes. We found a positive correlation between the expression of HSPB8 and BAG3 in ICC (Figure 1(c)). In addition, the expressions of HSPB8 and BAG3 in ICC cell lines (HCCC-9810 and RBE) were examined using qPCR and Western blot analysis. It was found that the expression of these proteins was upregulated compared to the intrahepatic bile duct epithelial cell line (Figure 1(d,e)). The experiments showed that HSPB8 and BAG3 were highly expressed in ICC and may play a significant role in tumor progression.

### BAG3 contributed to cell migration and invasion in ICC

**Figure 2.**
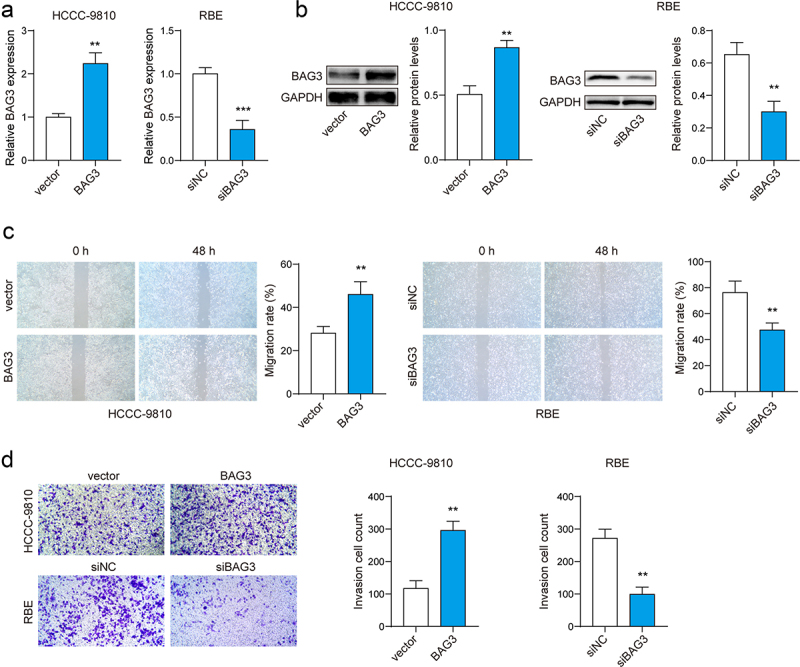
BAG3 contributed to ICC cell migration and invasion.

The overexpression of the BAG3 or silencing of the BAG3 plasmid was transfected into ICC cells to verify the impact of BAG3 on cell migration and invasion. The transfection was confirmed through by qPCR and Western blot analysis (Figure 2(a,b)). Then, we assessed the metastatic ability of ICC cells through a series of experiments. Wound healing assay demonstrated that overexpressing BAG3 enhanced the migration ability of cells, while silencing BAG3 inhibited cell migration (Figure 2(c)). The Transwell assay showed that overexpression of BAG3 enhanced cell invasion, whereas silencing BAG3 had the opposite effect (Figure 2(d)). In summary, the abnormally high expression of BAG3 facilitated the migration and invasion of ICC cells.

### HSPB8 promoted cellular autophagy mediated by BAG3

**Figure 3.**
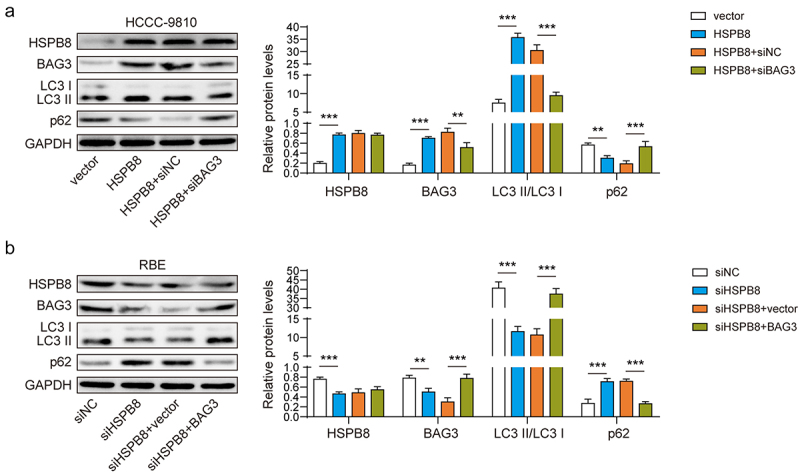
HSPB8 promoted cellular autophagy mediated by BAG3.

BAG3 has been shown to have an effect on cellular autophagy in previous studies,^14^ and HSPB8 functions as a chaperone that interacts with BAG3 during autophagy.^15^ Therefore, we explored the effect of HSPB8 and BAG3 on ICC cell autophagy. The experiments showed that overexpression of HSPB8 significantly increased the expression levels of BAG3 and LC3 (a protein widely used to monitor autophagy), but decreased the expression level of p62 (a multifunctional scaffold protein used to identify specific organelles and protein aggregates and transport them to autophagy for degradation^16^). This alteration could be partially reversed by silencing BAG3. In addition, silencing HSPB8 downregulated the expression of BAG3 and LC3 and upregulated the expression of p62, which was partially reversed by upregulating the level of BAG3 (Figure 3(a,b)). In short, HSPB8 could promote BAG3-mediated cellular autophagy in ICC cells.

### HSPB8-BAG3 degraded Filamin A by inducing autophagy

**Figure 4.**
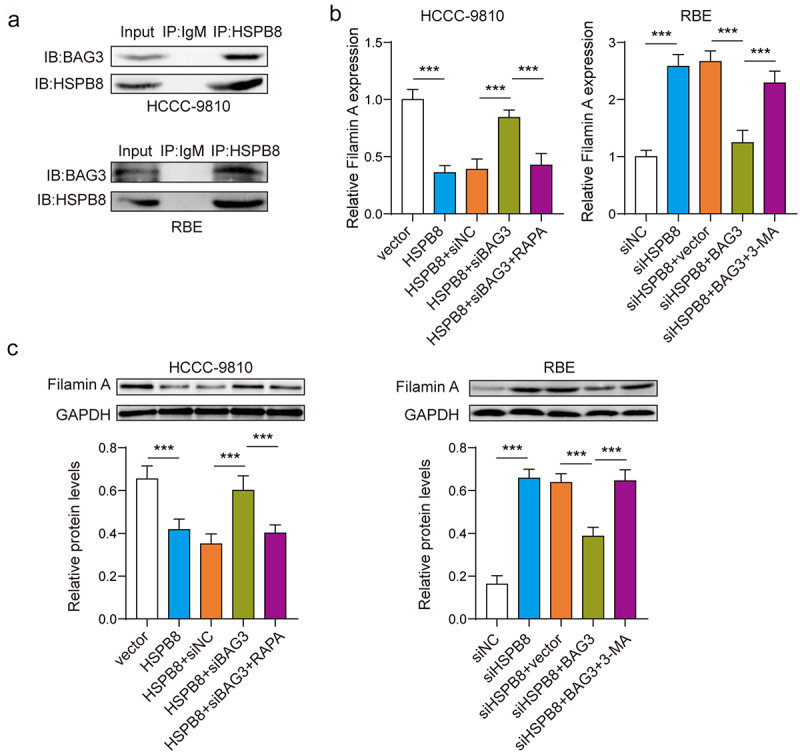
HSPB8-BAG3 degraded filamin A by inducing autophagy.

Subsequently, we performed Co-IP experiments using HSPB8 antibody and BAG3 antibody. The results demonstrated that the HSPB8 antibody pulled down the BAG3 protein and showed a band upon detection by the BAG3 antibody (Figure 4(a)). We then found that overexpression of HSPB8 downregulated Filamin A expression, and inhibiting BAG3 partially reversed this change. Conversely, silencing HSPB8 had the opposite effect, which was counteracted by upregulating BAG3. Meanwhile, activation of autophagy significantly decreased the expression levels of Filamin A, while inhibition of autophagy increased the expression levels of Filamin A (Figure 4(b,c)). Taken together, the HSPB8-BAG3 molecular chaperone complex can degrade Filamin A by inducing the autophagic process.

### Filamin A inhibited ICC cell migration and invasion

**Figure 5.**
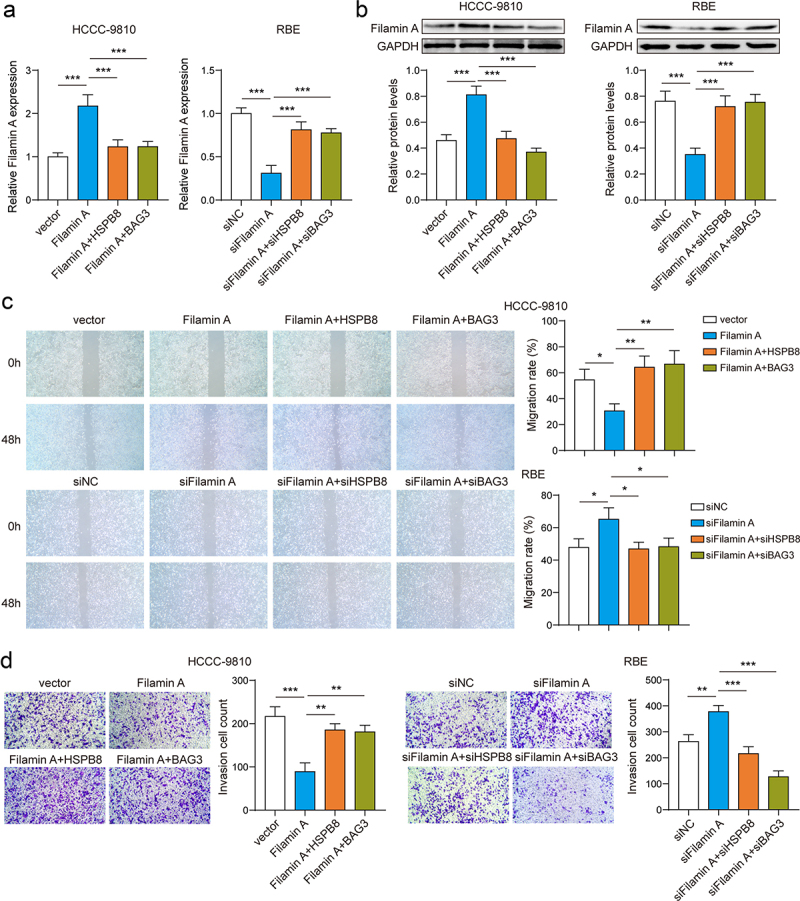
Filamin A inhibited ICC cell migration and invasion.

We investigated the impact of Filamin A on ICC cell migration and invasion. We verified the transfection efficiency of the Filamin A overexpression or silencing plasmid. Filamin A expression levels could be repressed by the overexpression of HSPB8 or BAG3 (Figure 5(a,b)). Overexpression of Filamin A inhibited the migration and invasion of ICC cells, but overexpression of HSPB8 or BAG3 could reverse this effect. Conversely, silencing Filamin A enhanced the metastatic ability of ICC cells, which could be reversed by silencing HSPB8 or BAG3 as well (Figure 5(c,d)). These results indicate that the HSPB8-BAG3 molecular chaperone complex promoted ICC cell migration and invasion by regulating CASA-mediated Filamin A degradation.

### Effects of HSPB8 and BAG3 on lung metastasis of tumors in vivo

**Figure 6.**
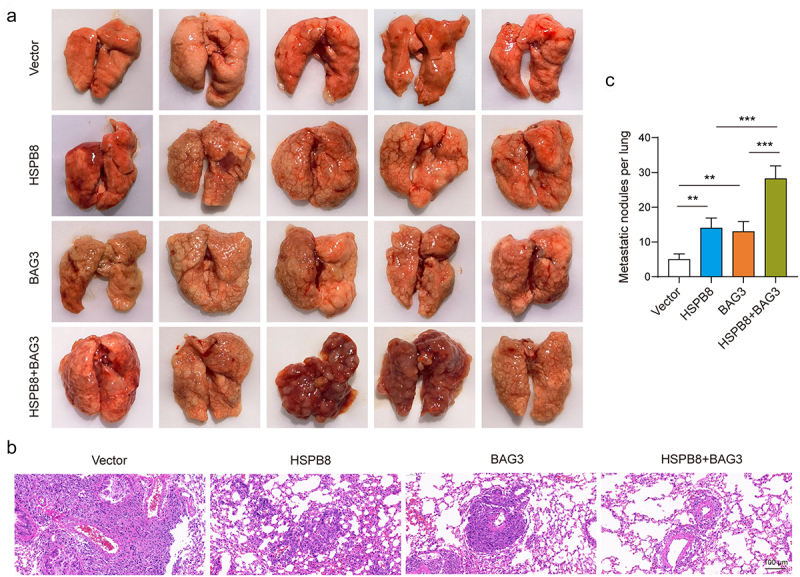
Effects of HSPB8 and BAG3 on lung metastasis of tumors *in vivo.*

Finally, we verified the effect of HSPB8 and BAG3 on lung metastasis of tumors by constructing a mouse metastasis model. Overexpression of HSPB8 and/or BAG3 significantly promoted lung metastasis of mouse tumors (Figure 6(a)). Further analysis of lung tissues revealed that the overexpression of HSPB8 and/or BAG3 led to increased immune infiltration in lung tissue and induced a higher number of metastatic nodules in the lungs (Figure 6(b-c)). These results suggest that HSPB8 and BAG3 could promote lung metastasis of ICC tumors in mice.

### Discussion

In recent years, ICC has garnered significant attention due to its escalating morbidity and mortality rates, without any enhancement in patient survival. Most patients are diagnosed with advanced cancer, and only a small number of them are eligible for surgery. However, the treatment of ICC still faces significant challenges due to the high recurrence rate and low survival rate after surgery.^17,18^ With the advancement of technology, researchers have begun to investigate the pathogenesis of ICC from a molecular perspective in an attempt to find effective diagnostic or therapeutic targets. However, no ideal target genes have been identified yet.^19^ In our study, we observed high expression levels of HSPB8 and BAG3 in ICC clinical samples. A series of molecular cellular experiments revealed that HSPB8 is positively correlated with the expression level of BAG3. Furthermore, HSPB8 was found to regulate the expression level of Filamin A during autophagy, thereby promoting the metastatic ability of ICC. This study reveals the mechanism by which the HSPB8-BAG3 molecular chaperone complex promotes ICC cell migration and invasion by regulating CASA-mediated Filamin A degradation. This finding provides a new perspective for the development of strategies for ICC diagnosis and treatment.

As one of the players in CASA, HSPB8 has been gradually confirmed to promote the development of various cancers. For example, HSPB8 is highly expressed in TNF-α-induced ovarian cancer and facilitates the migration of ovarian cancer cells.^6^ The researchers detected a higher expression level of HSPB8 in lung adenocarcinoma tissues and demonstrated that the elevated expression of HSPB8 promoted the proliferation and migration of lung adenocarcinoma cells.^20^ BAG3, a member of the Bcl-2 family, is a multifunctional accessory chaperone and anti-apoptotic protein. BAG3 is involved in the regulation of various physiological processes, including cellular stress response, apoptosis, and developmental regulation. However, abnormally expressed BAG3 can promote the malignant behavior of some cancers. In the development of colorectal cancer, thyroid cancer, and liver cancer, BAG3 can enhance the ability of cancer cells to metastasize, thereby complicating cancer treatment.^21^ Previous studies have shown that HSPB8 and BAG3 can combine to form a protein complex that regulates the CASA process, influencing cancer cell growth, migration, and apoptosis.^4^ However, there are few studies on the mechanism of action of the HSPB8-BAG3 complex on ICC progression. In our study, we found that both HSPB8 and BAG3 were upregulated in ICC tissues and cells, and their expression levels were positively correlated. Further experiments have shown that the upregulation of BAG3 expression enhances the migration and invasion of ICC cells, whereas the downregulation of BAG3 expression hinders cell migration and invasion. At the same time, overexpression of HSPB8 upregulated the levels of BAG3, and HSPB8 promotes BAG3-mediated autophagy.

Filamin A is an actin cross-linking protein that can bind to a variety of substances and participate in multiple cellular functions in the body. Among them, Filamin A has attracted much attention due to its crucial role in cell migration and invasion. Actually, Filamin A plays a dual role in cancer development. When Filamin A is localized in the cytoplasm, it promotes tumor development; however, when it is located in the nucleus, it hinders tumor development.^22^ In colorectal cancer and breast cancer, Filamin A plays a role in tumor development. Low expression is often positively correlated with the occurrence and development of diseases.^12,23^ In this study, increasing the expression of HSPB8 or BAG3, or activating autophagy, can suppress the expression of Filamin A. Furthermore, Filamin A could inhibit the migration and invasion of ICC cells, but this inhibition could be reversed by HSPB8 or BAG3. The follow-up will continue to combine clinical research to verify the relationship between the co-expression of HSPB8 and BAG3 and the prognosis of patients with ICC. This provides a new experimental foundation in the field of ICC research.

## Conclusion

Reducing the high morbidity and mortality of ICC is an urgent matter. On the basis that surgical interventions may not always achieve the desired curative effect, it is crucial to urgently develop and implement non-surgical treatment methods, such as drug therapy, that are effective. Molecular chaperones and co-chaperones could serve as potential targets for drug treatment of diseases.^24^ Based on this, we conducted experiments and discovered that the HSPB8-BAG3 chaperone complex can enhance ICC cell migration and invasion by regulating CASA-mediated degradation of Filamin A. This finding could potentially inspire the development of enhanced therapeutic strategies for ICC.

## Materials and methods

### Study subjects

ICC tissues (Tumor group) and normal tissues adjacent to the cancer (No-tumor group) were collected from 30 patients at the Second Xiangya Hospital of Central South University. All patients signed an informed consent form before the start of this study. The protocol of the study was approved by the Ethics Committee of The Second Xiangya Hospital, Central South University, and was in accordance with the ethical principles of the Declaration of Helsinki.

### Cell culture

The normal human intrahepatic bile duct cell line (HIBEC) was purchased from ATCC (USA), while the human intrahepatic cholangiocarcinoma cell lines (HCCC-9810, RBE) were purchased from iCell Bioscience Inc (Shanghai, China). DMEM containing 10% FBS and 1% penicillin-streptomycin (all from Gibco, USA) was used to culture all the cell lines separately at 37°C in a 5% CO_2_ humidified environment.^25^

### Transfection and drug treatment

For the overexpression of BAG3, HSPB8, and Filamin A, human BAG3, HSPB8, and Filamin A cDNA were PCR-amplified and then cloned into the expression vector pcDND3.1 (RiboBio, Guangzhou, China). For the silencing of BAG3, HSPB8, or Filamin A, small interfering RNAs (siRNAs) were purchased from GenePharma (Shanghai, China). Transfection was carried out using Lipofectamine 3000 (Invitrogen, Carlsbad, CA, USA), with the vector or siNC serving as the negative control. For autophagy activation or inhibition, cells were treated with RAPA (100 nM; Selleck Chemicals, Houston, TX, USA) or 3-MA (10 mM; Selleck Chemicals) for 48 hours.^26^

### Wound healing assay

A wound healing assay was performed to assess the migration ability of cells. Briefly, 1 × 10^6^ cells/well were seeded into a 6-well plate, and a 200 μL pipette tip was used to create scratches until the cells covered 90% of the bottom. At 0 and 48 hour after scratching, the wound area was measured and recorded.^27^

### Transwell assay

Cell invasion assay was conducted using Matrigel-coated inserts (BD Biosciences, Bedford, MA, USA). Serum-free medium (200 μL) was added to the upper chamber, and 800 μL of medium containing 10% FBS was added to the lower chamber. When the pre-work is ready, 5 × 10^4^ cells were seeded in the upper chamber. After 48 hours, the invaded cells in the lower chambers were fixed with 4% paraformaldehyde for 20 minutes and stained with 0.4% crystalline violet (Beyotime, Shanghai, China) for 20 minutes. The cells were then observed and counted under a microscope.^28^

### qPCR assay

Briefly, total RNA was extracted from the cells using the TRIzol RNA purification kit (Invitrogen, USA) and the reverse transcription reaction was performed with the PrimeScript™ II 1st strand cDNA synthesis kit (Takara, Japan). qPCR reactions were performed with SYBR Green PCR Premix (Applied Biosystems, USA).^29^ The results were expressed as relative expression levels calculated using the 2^−ΔΔCt^ method. The primers used in this experiment were shown in Table 1.

**Table 1.** Primer sequences for qPCR.

Gene	Primer sequences (5’-3’)
HSPB8	F: TCTCCAGAGGGTCTGCTCAT
	R: GCAGGTGACTTCCTGGTTGT
BAG3	F: CTCCATTCCGGTGATACACGA
	R: TGGTGGGTCTGGTACTCCC
Filamin A	F: AGCCTCCACGAGACATCATC
	R: CCAGTGTGTACTCCCCCTTG
GAPDH	F: AACGGATTTGGTCGTATTGGG
	R: CGCTCCTGGAAGATGGTGAT

### Western blot

The cells were centrifuged at 14,000 ×g for 15 minutes at 4°C, and the supernatant was collected for Western blot detection. The protein concentration was determined using the BCA protein assay kit (Beyotime). Proteins were separated by SDS-PAGE and transferred to PVDF membranes. Subsequently, the membranes were blocked with 5% nonfat milk at room temperature. Next, the membranes were incubated with the following primary antibodies: anti-HSPB8 (1:2000; ab151552), anti-BAG3 (1:5000; ab92309), anti-Filamin A (1:250000; ab76289), anti-LC3 (1:2000, ab192890), anti-p62 (1:10000; ab109012), and anti-GAPDH (1:10000, ab181602) overnight at 4°C. After washing, the membranes were incubated with the secondary antibody (1:2000; ab6702) for 1 hour at room temperature. The protein bands were visualized by incubation with enhanced chemiluminescent (ECL) reagent (Solarbio, Beijing, China), and ImageJ software was utilized to quantify the intensity of the protein bands. All antibodies were purchased from Abcam (USA).^30^

### Co-IP assay

Cells were lysed on the plate using RIPA lysis buffer for 30 minutes. In addition, 50 μL of Dynabeads Protein G (Sigma-Aldrich, USA) was incubated with the antibody for 1 hour at room temperature. The cell lysate was thoroughly mixed with the magnetic beads-antibody complex and incubated overnight at 4°C. The samples were washed three times with lysis buffer, and the bound protein complexes were detected by Western blotting.^31^

### Lung metastasis in vivo

BALB/c male nude mice (5 weeks old) were purchased from the Guangdong Medical Experimental Animal Center (China). HCCC-9810 cells (2 × 10^6^) overexpressing HSPB8 and/or BAG3 were injected into mice (five mice per group) via the tail vein. After 60 days, the mice were euthanized via spinal dislocation, and their lungs were surgically removed to capture images and quantify the number of nodules. Then, the lung tissues of mice were fixed in 10% formalin and embedded in paraffin for hematoxylin and eosin (H&E) staining to adhere histology. All animal operations conform to ethical standards and have been approved by the Ethics Committee of The Second Xiangya Hospital, Central South University.

### Statistical analysis

All experiments were repeated three times, and the experimental data were presented as the mean ± standard deviation. Student’s *t*-test or ANOVA followed by Tukey’s post hoc test was performed for comparisons between two groups or multiple groups. Pearson correlation analysis was used to examine the correlation between HSPB8 and BAG3 expression levels in clinical samples. Data were statistically significant when *p* < .05.

## Highlights

- The expressions of HSPB8 and BAG3 were upregulated in ICC tissues and cells, and their expressions were positively correlated.
- The metastatic ability of ICC cells could be promoted or inhibited by upregulating or downregulating the expression of BAG3.
- The HSPB8-BAG3 chaperone complex led to the abnormal degradation of Filamin A by activating autophagy.
- The increased expression of Filamin A inhibited the migration and invasion of ICC cells.

## Abbreviation

ICC: intrahepatic cholangiocarcinoma
HSPB8: heat shock protein B8
CASA: chaperone-assisted selective autophagy
EMT: epithelial-mesenchymal transition
BAG3: Bcl-2-associated athanogene 3
ATCC: American Type Culture Collection
DMEM: Dulbecco’s Modified Eagle’s medium
FBS: fetal bovine serum
qPCR: quantitative real-time PCR
GAPDH: glyceraldehyde-3phosphate dehydrogenase
RIPA: radio-immunoprecipitation assay
BCA: bicinchoninic acid
Co-IP: co-immunoprecipitation
ANOVA: analysis of variance

## Data Availability

The datasets used or analyzed during the current study are available from the corresponding author on reasonable request.
